# Social Inequalities and Mortality in Europe – Results from a Large Multi-National Cohort

**DOI:** 10.1371/journal.pone.0039013

**Published:** 2012-07-25

**Authors:** Valentina Gallo, Johan P. Mackenbach, Majid Ezzati, Gwenn Menvielle, Anton E. Kunst, Sabine Rohrmann, Rudolf Kaaks, Birgit Teucher, Heiner Boeing, Manuela M. Bergmann, Anne Tjønneland, Susanne O. Dalton, Kim Overvad, Maria-Luisa Redondo, Antonio Agudo, Antonio Daponte, Larraitz Arriola, Carmen Navarro, Aurelio Barricante Gurrea, Kay-Tee Khaw, Nick Wareham, Tim Key, Androniki Naska, Antonia Trichopoulou, Dimitrios Trichopoulos, Giovanna Masala, Salvatore Panico, Paolo Contiero, Rosario Tumino, H. Bas Bueno-de-Mesquita, Peter D. Siersema, Petra P. Peeters, Sophia Zackrisson, Martin Almquist, Sture Eriksson, Göran Hallmans, Guri Skeie, Tonje Braaten, Eiliv Lund, Anne-Kathrin Illner, Traci Mouw, Elio Riboli, Paolo Vineis

**Affiliations:** 1 Department of Epidemiology and Biostatistics, School of Public Health, Imperial College London, London, United Kingdom; 2 Social and Environmental Health Research, London School of Hygiene and Tropical Medicine, London, United Kingdom; 3 Department of Public Health, Erasmus Medical Centre, Rotterdam, The Netherlands; 4 Epidemiology of Occupational and Social Determinants of Health, INSERM, Villejuif, France; 5 Université of Versailles Saint Quentin, Versailles, France; 6 Department of Public Health, Academic Medical Centre, University of Amsterdam, Amsterdam, The Netherlands; 7 Division of Cancer Epidemiology and Prevention, Institute of Social and Preventive Medicine, University of Zurich, Zurich, Switzerland; 8 Division of Clinical Epidemiology, German Cancer Research Centre, Heidelberg, Germany; 9 Institute of Human Nutrition, Potsdam-Rehbrücke, Germany; 10 Institute of Cancer Epidemiology, Danish Cancer Society, Copenhagen, Denmark; 11 Department of Clinical Epidemiology, Aarhus University Hospital, Aarhus, Denmark; 12 Public Health and Participation Directorate, Health and Health Care Services Council, Oviedo, Asturias, Spain; 13 Unit of Nutrition, Environment and Cancer, Catalan Institute of Oncology, Barcelona, Spain; 14 CIBER Epidemiologia y Salud Publica, Barcelona, Spain; 15 Andalusian School of Public Health, Granada, Spain; 16 Public Health Division of Gipuzkoa, Basque Government, Spain; 17 Department of Epidemiology, Regional Health Authority, Murcia, Spain; 18 Departamento de Ciencias Sociosanitarias, University of Murcia School of Medicine, Murcia, Spain; 19 Navarra Public Health Institute, Pamplona, Spain; 20 University of Cambridge, Cambridge, United Kingdom; 21 Medical Research Council – Epidemiology Unit, Cambridge, United Kingdom; 22 Cancer Epidemiology Unit, University of Oxford, Oxford, United Kingdom; 23 World Health Organisation Collaborating Centre for Food and Nutrition Policies, Department of Hygiene, Epidemiology and Medical Statistics, University of Athens Medical School, Athens, Greece; 24 Hellenic Health Foundation, Athens, Greece; 25 Department of Epidemiology, Harvard School of Public Health, Boston, United States of America; 26 Bureau of Epidemiologic Research, Academy of Athens, Greece; 27 Molecular and Nutritional Epidemiology Unit, Cancer Research and Prevention Institute, Florence, Italy; 28 Department of Clinical and Experimental Medicine, Federico II University, Naples, Italy; 29 Cancer Registry and Environmental Epidemiology, Istituto Nazionale Tumori, Milan, Italy; 30 Cancer Registry and Histopathological Unit, Civile M. P. Arezzo Hospital, Ragusa, Italy; 31 National Institute for Public Health and the Environment, Bilthoven, The Netherlands; 32 Department of Gastroenterology and Hepatology, University Medical Centre Utrecht, Utrecht, The Netherlands; 33 Julius Center for Health Sciences and Primary Care, University Medical Center Utrecht, Utrecht, Netherlands; 34 Department of Clinical Sciences in Malmö, Diagnostic Radiology, Lund University, Sweden; 35 Department of Surgery, University Hospital Lund and Lund University, Lund, Sweden; 36 Department of Community Medicine and Rehabilitation, Umeå University, Umeå, Sweden; 37 Institutt for samfunnsmedisin, Universitetet i Tromsø, Tromsø, Norway; 38 Dietary Exposure Assessment Group, Nutrition and Metabolism Section, International Agency for Research on Cancer, Lyon, France; University of Bochum, Germany

## Abstract

**Background:**

Socio-economic inequalities in mortality are observed at the country level in both North America and Europe. The purpose of this work is to investigate the contribution of specific risk factors to social inequalities in cause-specific mortality using a large multi-country cohort of Europeans.

**Methods:**

A total of 3,456,689 person/years follow-up of the European Prospective Investigation into Cancer and Nutrition (EPIC) was analysed. Educational level of subjects coming from 9 European countries was recorded as proxy for socio-economic status (SES). Cox proportional hazard model's with a step-wise inclusion of explanatory variables were used to explore the association between SES and mortality; a Relative Index of Inequality (RII) was calculated as measure of relative inequality.

**Results:**

Total mortality among men with the highest education level is reduced by 43% compared to men with the lowest (HR 0.57, 95% C.I. 0.52–0.61); among women by 29% (HR 0.71, 95% C.I. 0.64–0.78). The risk reduction was attenuated by 7% in men and 3% in women by the introduction of smoking and to a lesser extent (2% in men and 3% in women) by introducing body mass index and additional explanatory variables (alcohol consumption, leisure physical activity, fruit and vegetable intake) (3% in men and 5% in women). Social inequalities were highly statistically significant for all causes of death examined in men. In women, social inequalities were less strong, but statistically significant for all causes of death except for cancer-related mortality and injuries.

**Discussion:**

In this European study, substantial social inequalities in mortality among European men and women which cannot be fully explained away by accounting for known common risk factors for chronic diseases are reported.

## Introduction

Reducing health inequalities between and within countries is an important challenge for health policy [1], [2]. Higher mortality rates among subjects with lower socioeconomic position are observed at country level in both North America and Europe [2]–[6]. Such differences have been reported for most causes of death, in particular for cardiovascular diseases and some types of cancer. Although some of the differences are due to treatment and survival being unevenly distributed across social classes, differences in incidence rates for the underlying conditions seem to play a major role [7]–[9]. A better understanding of the mechanisms underlying these inequalities will help defining the most effective preventive policies. To identify the intermediate (e.g. behavioural, environmental, or biological) factors that explain these inequalities is a first step to uncovering these mechanisms.

A descriptive study, aimed at measuring variations in the magnitude of inequalities in health among 22 European countries and at identifying some of the specific determinants of these variations, was recently published [3]. In almost all countries the rates of death were reported as substantially higher in groups of lower socioeconomic status and the magnitude of the inequalities was much larger in some countries than in others, and this was somewhat greater for cardiovascular diseases than for cancer-related mortality [3]. Although these survey data suggested that smoking, excessive alcohol consumption and obesity contributed to these inequalities they do not allow disentangling the association between socioeconomic status, specific determinants, and mortality at the individual level. In order to further investigate this complex interplay, an analysis of a large European prospective study which includes extensive and detailed information on risk factors at the individual level was undertaken with the purpose of investigating the contribution of specific intermediate determinants to social inequalities in cause-specific mortality.

**Table 1:** Demographic characteristics of the sample, and smoking status, alcohol consumption, physical activity, and BMI at recruitment in men.

Men	None or Primary school N = 49,979	Technical/Professional school N = 33,473	Secondary school N = 17,565	University N = 32,276	All N = 133,293
Mean age (SD)	55.7 (8.5)	52.1 (9.4)	48.1 (10.9)	51.5 (8.9)	52.8 (20.1)
Total deaths (%)	4,618 (9.2)	2,164 (6.5)	736 (4.2)	1,495 (4.6)	9,013 (6.8)
Alcohol consumption, g/day (median, IQR)	12.9 (3.0–33.6)	13.2 (4.4–30.2)	11.7 (3.7–26.8)	16.3 (6.7–32.7)	13.6 (4.2–31.5)
Alcohol abstainers, (%)	5,094 (10.2)	2,127 (6.4)	993 (5.7)	1,421 (4.4)	9,632 (7.2)
Body Mass index					
Underweight (%)	156 (0.3)	120 (0.4)	85 (0.5)	113 (0.4)	474 (0.4)
Normal weight (%)	12,857 (25.7)	11,524 (34.4)	7,337 (41.8)	13,124 (40.7)	44,842 (33.6)
Overweight (%)	25,933 (51.9)	16,962 (50.7)	8,064 (45.9)	15,439 (47.8)	66,398 (49.8)
Obese (%)	11,033 (22.1)	4,867 (14.5)	2,079 (11.8)	3,600 (11.2)	21,579 (16.2)
Smoking status					
Never (%)	13,638 (27.3)	9,834 (29.4)	6,474 (36.9)	11,930 (37.0)	41,876 (31.4)
Former (%)	18,806 (37.6)	12,884 (38.5)	5,892 (33.5)	12,076 (37.4)	49,658 (37.3)
Current (%)	17,535 (35.1)	10,755 (32.1)	5,199 (29.6)	8,270 (25.6)	41,759 (31.1)
Leisure physical activity					
Inactive (%)	16,604 (33.2)	10,254 (30.6)	5,814 (33.1)	10,840 (33.6)	43,512 (32.6)
Moderately active (%)	15,896 (31.8)	10,850 (32.4)	5,804 (33.0)	11,123 (34.5)	43,673 (32.8)
Active (%)	17,149 (34.3)	11,343 (33.9)	5,485 (31.2)	9,741 (30.2)	43,718 (32.8)
Undetermined (%)	330 (0.7)	1,026 (3.1)	462 (2.6)	572 (1.8)	2,390 (1.8)
Country					
Italy	6,131 (42.8)	2,142 (15.0)	4,059 (28.3)	1,989 (13.9)	14,321 (100.00)
Spain	9.982 (64.2)	1,991 (12.8)	1,248 (8.0)	2,318 (14.9)	15,539 (100.00)
UK	3,255 (32.2)	4,434 (43.9)	1,033 (10.2)	1,374 (13.6)	10.096 (100.00)
Netherlands	1,086 (10.8)	4,191 (41.7)	2,083 (20.7)	2,687 (26.7)	10,047 (100.00)
Greece	5,825 (56.2)	1,521 (14.7)	1,032 (10.0)	1,992 (12.2)	10,370 (100.00)
Germany	5,686 (25.0)	6,297 (27.7)	1,184 (5.2)	9,596 (42.2)	22,763 (100.00)
Sweden	8,599 (37.2)	4,992 (21.6)	4,836 (20.9)	4,721 (20.4)	23,148 (100.00)
Denmark	9,415 (34.9)	7,905 (29.3)	2,090 (7.7)	7,599 (28.1)	27,009 (100.00)
Norway	-	-	-	-	-

IQR =  inter quartile range.

**Table 2:** Demographic characteristics of the sample, and smoking status, alcohol consumption, physical activity, and BMI at recruitment in women.

Women	None or Primary school N = 93,058	Technical/Professional school N = 65,999	Secondary school N = 41,119	University N = 37,826	All N = 238,002
Mean age (SD)	54.2 (8.9)	51.5 (8.7)	48.5 (9.5)	48.3 (8.9)	51.5 (9.3)
Total deaths (%)	3,552 (3.8)	1,889 (2.9)	833 (2.0)	685 (1.8)	6,959 (2.9)
Alcohol consumption, g/day (median, IQR)	1.3 (0–7.0)	3.7 (1.0–10.8)	3.0 (0.6–10.0)	5.2 (1.2–12.5)	2.6 (0.4–9.7)
Alcohol abstainers (%)	28,703 (30.8)	8,338 (12.6)	6,393 (15.6)	4,370 (11.6)	47,804 (20.1)
Body Mass index					
Underweight (%)	709 (0.8)	898 (1.4)	668 (1.6)	752 (2.0)	3,027 (1.3)
Normal weight (%)	32,398 (34.8)	35,677 (54.1)	24,714 (60.1)	24,403 (64.5)	117,192 (49.2)
Overweight (%)	36,415 (39.1)	21,290 (32.3)	11,907 (29.0)	9,746 (25.8)	79,358 (33.3)
Obese (%)	23,536 (25.3)	8,134 (12.3)	3,830 (9.3)	2,952 (7.7)	38,425 (16.1)
Smoking status					
Never (%)	55,753 (59.9)	29,841 (45.2)	18,628 (45.3)	18,722 (49.5)	122,944 (51.7)
Former (%)	15,597 (16.8)	17,717 (26.8)	11,379 (27.7)	10,814 (28.6)	55,507 (23.3)
Current (%)	21,708 (23.3)	18,441 (27.9)	11,112 (27.0)	8,290 (21.9)	59,551 (25.0)
Leisure physical activity					
Inactive (%)	27,209 (29.2)	21,150 (32.1)	14,437 (35.1)	15,332 (40.5)	78,128 (32.8)
Moderately active (%)	30,896 (33.2)	21,751 (33.0)	13,555 (33.0)	12,125 (32.1)	78,327 (32.9)
Active (%)	34,407 (27.0)	22,005 (33.3)	12,292 (29.9)	9,878 (26.1)	78,582 (33.0)
Undetermined (%)	546 (0.6)	1,093 (1.7)	835 (2.0)	491 (1.3)	2,965 (1.3)
Country					
Italy	16,790 (52.5)	3,529 (11.0)	7,387 (23.1)	4,294 (13.3)	31,955 (100.00)
Spain	20,319 (79.4)	1,405 (5.5)	1,428 (5.6)	2,445 (9.6)	25,597 (100.00)
UK	5,477 (45.4)	4,434 (36.7)	833 (6.9)	1,323 (11.0)	12,067 (100.00)
Netherlands	5,186 (18.3)	9,354 (33.0)	8,674 (30.6)	5,134 (18.1)	28,248 (100.00)
Greece	10,195 (67.9)	469 (3.1)	2,156 (14.4)	2,199 (14.6)	15,019 (100.00)
Germany	7,220 (23.9)	12,563 (41.6)	2,394 (7.9)	7,993 (26.5)	30,170 (100.00)
Sweden	10,323 (34.4)	7,840 (26.1)	4,778 (15.9)	7,094 (23.6)	30,035 (100.00)
Denmark	9,366 (31.5)	13,806 (46.5)	3,504 (11.8)	3,043 (10.2)	29,719 (100.00)
Norway	93,058 (39.1)	65,999 (27.7)	41,119 (17.3)	37,826 (15.9)	238,002 (100.00)

IQR =  inter quartile range.

## Methods

### Population

The European Prospective Investigation into Cancer and Nutrition (EPIC) is a multicenter prospective cohort recruiting more than 520,000 persons in years 1992–1998, mostly aged 40 to 65 years. In the present analysis, participants recruited in Norway, Sweden (Malmö, Umea), Denmark (Copenhagen, Aarhus), The Netherlands (Utrecht, Bilthoven), Great Britain (Cambridge), Germany (Potsdam, Heidelberg), France, Italy (Florence, Varese, Ragusa, Turin, and Naples), Spain (Asturias, Granada, Murcia, Navarra, and San Sebastian), and Greece were included. In most centres, subjects were recruited from the general population in a given geographic area; some Spanish and Italian centres included blood donors; the Utrecht cohort was based on women participating in a mammography screening program; the cohorts in Norway, Utrecht, and Naples include women only. Although some sub-cohorts were not selected directly from the general population, this does not affect internal comparisons which remain valid. At the time of enrolment, all subjects completed a dietary and lifestyle questionnaire including questions on smoking status and intensity, alcohol consumption, leisure physical activity, and fruit and vegetable consumption [10]. Information on fruit, vegetables, and alcohol consumption was obtained via a Food Frequency Questionnaire enquiring about diet and alcoholic beverage consumption in the previous 12 months; this information was calibrated on a 24 h dietary recall interview generating detailed information in food and beverages consumed during the day before the interview resulting in an estimated consumption of fruit, vegetables, and alcohol in g/day 10,11. Information on physical activity was derived by a combination of an index of self-reported physical activity at work (sedentary, standing, manual, heavy manual) and the sum of time spent cycling or performing any other sport (none, ≤3.5 hrs/wk, >3.5 and ≤7 hrs/wk, >7 hrs/wk) [12]. Anthropometric characteristics were also measured at enrolment.

**Figure 1:**
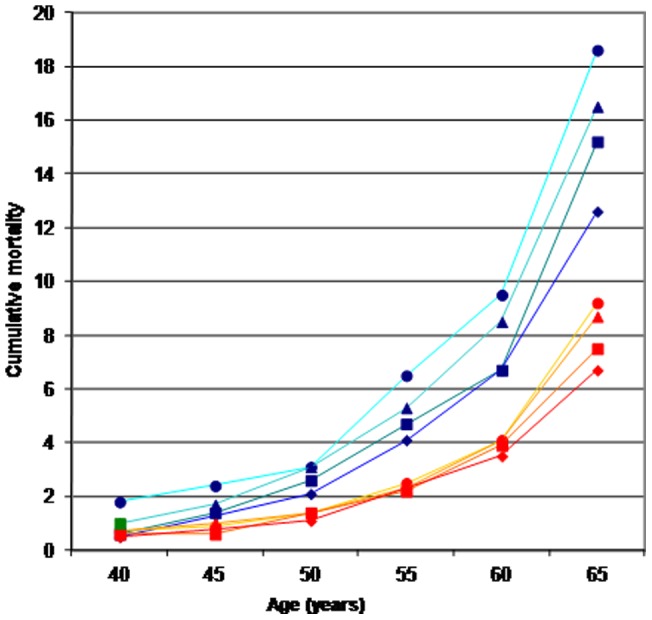
Cumulative mortality at different ages by education level and sex (blue lines for men, orange/red lines for women; circles for none-primary education, triangles for technical education, squares for secondary education, diamonds for university degree).

Individuals with missing information on educational level (N = 5,697; 1.5%), smoking status (N = 4,741; 1.2%), and alcohol consumption (N = 4,674; 1.2%) were excluded from the analysis which was based on 371,295 participants. In total, 9,892 men (7.4% of men) and 7,791 women (3.3% of women) died in 3,456,689 person/years follow-up.

**Table 3:** Cox regression-derived Hazard Ratios (HR) for total mortality across educational levels and across Relative Inequality Index (RII) in crude (Model 1) and adjusted (Model 2 to 4) models.

		N	Person/years	Mortality (%)	Model 1 HR*	95% C.I.	p-value	Model 2 HR†	95% C.I.	p-value	Model 3 HR‡	95% C.I.	p-value	Model 4 HR**	95% C.I.	p-value	Model 4a†† HR**	95% C.I.	p-value
**Men**		**133,293**															**41,876**		
Educational level																			
None/primary		49,979	447,745	4,618 (9.2)	Ref.	-	-	Ref.	-	-	Ref.	-	-	Ref.	-	-	Ref.	-	-
Technical		33,473	303,793	2,164 (6.5)	0.84	0.79–0.88	<0.001	0.86	0.82–0.91	<0.001	0.87	0.82–0.92	<0.001	0.89	0.84–0.94	<0.001	0.91	0.80–1.04	0.167
Secondary		17,565	157,645	736 (4.2)	0.76	0.70–0.82	<0.001	0.79	0.73–0.86	<0.001	0.80	0.74–0.87	<0.001	0.83	0.76–0.90	<0.001	0.86	0.72–1.03	0.111
University		32,276	286,863	1,495 (4.6)	0.66	0.62–0.70	<0.001	0.72	0.68–0.77	<0.001	0.74	0.70–0.79	<0.001	0.77	0.72–0.82	<0.001	0.78	0.67–0.90	0.001
					Trend		<0.001		Trend	<0.001		Trend	<0.001		Trend	<0.001		Trend	0.001
RII					0.57	0.52–0.61	<0.001	0.64	0.59–0.69	<0.001	0.66	0.60–0.71	<0.001	0.69	0.64–0.75	<0.001	0.72	0.59–0.87	0.001
**Women**		**238,002**															**122,944**		
Educational level																			
None/primary		93,058	802,715	3,552 (3.8)	Ref.	-	-	Ref.	-	-	Ref.	-	-	Ref.	-	-	Ref.	-	-
Technical		65,999	535,049	1,889 (2.9)	0.84	0.79–0.90	<0.001	0.87	0.82–0.92	<0.001	0.88	0.83–0.94	<0.001	0.90	0.85–0.96	0.001	1.03	0.93–1.14	0.532
Secondary		41,119	319,505	833 (2.0)	0.86	0.79–0.93	<0.001	0.87	0.81–0.95	0.001	0.90	0.83–0.97	0.009	0.93	0.85–1.01	0.067	1.12	0.98–1.27	0.100
University		37,826	313,025	685 (1.8)	0.75	0.69–0.82	<0.001	0.78	0.72–0.85	<0.001	0.81	0.74–0.88	<0.001	0.84	0.77–0.92	<0.001	0.90	0.78–1.04	0.152
					Trend		<0.001		Trend	<0.001		Trend	<0.001		Trend	<0.001		Trend	0.657
RII					0.71	0.64–0.78	<0.001	0.75	0.68–0.82	<0.001	0.78	0.71–0.86	<0.001	0.83	0.75–0.92	<0.001	1.04	0.88–1.22	0.657
**Men and women**		**371,295**															**164,820**		
Educational level																			
None/primary		143,037	1,250,46	8,170 (5.7)	Ref.	-	-	Ref.	-	-	Ref.	-	-	Ref.	-	-	Ref.	-	-
Technical		99,472	838,841	4,053 (4.1)	0.84	0.81–0.88	<0.001	0.86	0.83–0.90	<0.001	0.87	0.84–0.91	<0.001	0.89	0.86–0.93	<0.001	0.99	0.91–1.07	0.794
Secondary		58,684	477,151	1,569 (2.7)	0.81	0.76–0.85	<0.001	0.83	0.79–0.88	<0.001	0.85	0.80–0.90	<0.001	0.87	0.83–0.93	<0.001	1.02	0.92–1.14	0.663
University		70,102	599,888	2,180 (3.1)	0.69	0.66–0.73	<0.001	0.75	0.71–0.79	<0.001	0.76	0.73–0.80	<0.001	0.79	0.75–0.83	<0.001	0.85	0.77–0.94	0.001
					Trend		<0.001		Trend	<0.001		Trend	<0.001		Trend	<0.001		Trend	0.008
RII					0.62	0.59–0.66	<0.001	0.69	0.65–0.73	<0.001	0.71	0.67–0.76	<0.001	0.75	0.70–0.80	<0.001	0.90	0.79–1.02	0.085

* including sex and stratified by centre of recruitment and age; †including sex, smoking status at recruitment (never smoker, former smoker ≥10 years, former smoker <10 years, former smoker unknown, current smoker <15 cigarettes/day, 15–24 cigarettes/day, ≥25 cigarettes/day) and stratified by centre of recruitment; **‡**including sex, smoking status at recruitment (as in †) and BMI in 2.5 kg/m^2^ categories (<20.0; 20.1–22.5; 22.6–25.0; 25.1–22.5; 22.6–30.0; 30.1–32.5; 32.6–35.0; 35.1–37.5; ≥37.6) and stratified by centre of recruitment; ** including sex, smoking status at recruitment and BMI (as in ‡) and alcohol consumption at recruitment (g/day, in deciles of distribution), leisure physical activity (inactive, moderately active, active, and unknown), and fruit and vegetables consumption; ††never smoker only.

Information about the highest attained educational level was collected using country-specific questionnaires and classified as primary education or less, technical or professional education, secondary education, and college or university. These correspond to the UNESCO Standard Classification of Education-Attainment (ISCED-A) class 0–1 (less than primary, and primary), 2 (lower secondary), 3 (upper secondary), and 4–8 (post-secondary non-tertiary, short-cycle tertiary, Bachelor or equivalent, Master or equivalent, and Doctoral or equivalent) [13]. The analysis is based on education as a proxy for socio-economic status (SES). The study has been approved by the IARC and Imperial College Ethics Committees and by all the local Ethics Committees (The Committee of Bioethics and Deontology of the Hellenic Health Foundation, Athens, Greece; Norwich District Ethics Committee, Cambridge, UK; The National Committee on Health Research Ethic, Denmark; Comité de Protection des Personnes, France; Ethics Committee of the Heidelberg University Medical School, Germany; Comitato Etico Indipendente, Fondazione IRCCS Istituto Nazionale dei Tumori, Milan, Italy; Comitato Etico Locale Azienda Sanitaria di Firenze, Florence, Italy; Ethics Committee of Lundst University, Malmo, Sweden; The Medical Ethical Committee (METC =  Medisch Ethische Toetsingscommissie) of the University Medical Center Utrecht (UMCU), Utrecht, the Netherlands; The Regional Committee for Medical and Health Research Ethics, North Norway; Scotland A Research Ethics Committee, Oxford, UK; Ethikkommission der Landesärztekammer Brandenburg Cottbus, Germany; CEIC Comité de Ética de Investigación Clínica, Spain; Human Genetics Foundation Torino: Ethics Committee, Turin, Italy; Umea Regional Ethical Review Board, Sweden); all participants gave their written informed consent to take part in the study.

**Table 4:** Relative Index of Inequality (RII) for specific causes of death in men and women.

Cancer-related mortality		Model 1 HR*	95% C.I.	p-value	Model 2 HR†	95% C.I.	p-value	Model 3 HR‡	95% C.I.	p-value	Model 4 HR**	95% C.I.	p-value	Never smokers	Model 4a HR** ‡‡	95% C.I.	p-value
Men	3,072 (2.3)	0.68	0.59–0.78	<0.001	0.76	0.66–0.88	<0.001	0.76	0.66–0.87	<0.001	0.77	0.67–0.89	<0.001	603 (1.4)	0.77	0.55–1.06	0.111
Women	3,241 (1.4)	0.93	0.80–1.07	0.282	0.95	0.83–1.10	0.516	0.99	0.86–1.14	0.875	0.99	0.86–1.14	0.892	1,534 (1.3)	1.03	0.89–1.19	0.706
**Breast cancer death**																	
Men	4 (0.0)	-	-	-	-	-	-	-	-	-	-	-	-	2 (0.0)	-	-	-
Women	552 (0.2)	0.92	0.65–1.29	0.625	0.93	0.66–1.30	0.661	1.05	0.74–1.48	0.790	1.03	0.72–1.46	0.880	298 (0.2)	1.55	0.94–2.57	0.084
**Lung cancer death††**																	
Men	755 (0.6)	0.31	0.23–0.41	<0.001	0.49	0.33–0.73	0.001	0.46	0.30–0.69	<0.001	0.47	0.31–0.71	<0.001	27 (0.1)	0.31	0.06–1.66	0.171
Women	453 (0.2)	0.61	0.42–0.88	0.008	0.80	0.50–1.28	0.354	0.75	0.47–1.21	0.239	0.86	0.53–1.39	0.533	79 (0.1)	0.42	0.14–1.25	0.118
**All cardiovascular death**																	
Men	2,663 (2.0)	0.49	0.42–0.58	<0.001	0.55	0.47–0.64	<0.001	0.58	0.50–0.68	<0.001	0.65	0.56–0.76	<0.001	490 (1.2)	0.61	0.42–0.89	0.010
Women	1,488 (0.6)	0.49	0.39–0.61	<0.001	0.50	0.40–0.63	<0.001	0.56	0.44–0.70	<0.001	0.62	0.49–0.78	<0.001	710 (0.6)	0.72	0.50–1.05	0.091
**IHD death**																	
Men	1,471 (1.1)	0.43	0.35–0.53	<0.001	0.48	0.39–0.59	<0.001	0.51	0.41–0.63	<0.001	0.58	0.46–0.71	<0.001	275 (0.7)	0.64	0.39–1.04	0.073
Women	533 (0.2)	0.42	0.28–0.61	<0.001	0.43	0.29–0.62	<0.001	0.49	0.34–0.72	<0.001	0.61	0.41–0.90	0.012	234 (0.2)	0.97	0.52–1.84	0.932
**Cerebrovascular death**																	
Men	407 (0.3)	0.58	0.34–0.88	0.010	0.62	0.41–0.94	0.025	0.64	0.42–0.96	0.032	0.72	0.47–1.09	0.119	99 (0.2)	0.51	0.21–1.20	0.123
Women	448 (0.2)	0.59	0.39–0.89	0.012	0.60	0.40–0.91	0.015	0.62	0.41–0.94	0.023	0.65	0.43–0.99	0.044	236 (0.2)	0.75	0.39–1.45	0.395
**Injuries**																	
Men	301 (0.2)	0.56	0.35–0.90	0.016	0.61	0.38–0.97	0.036	0.60	0.38–0.96	0.033	0.61	0.38–0.98	0.041	81 (0.2)	0.79	0.31–2.01	0.624
Women	167 (0.1)	1.28	0.67–2.45	0.450	1.27	0.67–2.41	0.467	1.09	0.57–2.08	0.802	1.19	0.61–2.30	0.609	86 (0.1)	3.32	1.27–8.68	0.014

* stratified by centre of recruitment and age; †including smoking status at recruitment (never smoker, former smoker ≥10 years, former smoker <10 years, former smoker unknown, current smoker <15 cigarettes/day, 15–24 cigarettes/day, ≥25 cigarettes/day) and stratified by centre of recruitment; ‡ including smoking status at recruitment (as in †) and BMI in 2.5 kg/m^2^ categories (<20.0; 20.1–22.5; 22.6–25.0; 25.1–22.5; 22.6–30.0; 30.1–32.5; 32.6–35.0; 35.1–37.5; ≥37.6) and stratified by centre of recruitment; ** including smoking status at recruitment and BMI (as in ‡) and alcohol consumption at recruitment (g/day, in deciles of distribution), leisure physical activity (inactive, moderately active, active, and unknown), and fruit and vegetables consumption; ††models including smoking are adjusted for smoking status at recruitment as a categorical variable (never, current, or former smoker); age at the start of, and duration of, smoking (in years) as continuous variables; a linear and a quadratic term for current quantity smoked (number of cigarettes per day); and two interaction terms between duration and quantity and between age at start and duration; ‡‡ never smoker only.

**Figure 2:**
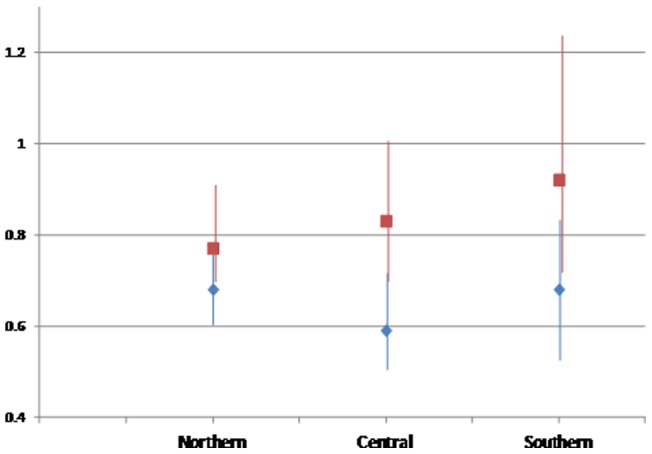
Hazard ratio (HRs) for mortality across the Relative Index of Inequality (RII) in men (blue diamonds) and women (red squares), in Northern (Norway, Sweden, Denmark), Central (UK, Netherlands, and Germany), and Southern (Spain, Italy, and Greece) European countries; fully adjusted model (including smoking status at recruitment, BMI in 2.5 kg/m^2^ categories, alcohol consumption at recruitment, leisure physical activity, and fruit and vegetables consumption, and stratifyied by age and centre of recruitment).

### End points

The follow-up period considered for this analysis ended between December 2002 and December 2006, the mean follow-up period being 9.3 years (SD 2.3). The outcome variable was death from any cause. Causes of death have been coded according to International Classification of Disease- 10^th^ revision (ICD-10); grouping of specific causes of death aimed at maximising consistency with previous works [3], [14]: cancer-related deaths were defined as those deaths whose underlying cause of death was coded ICD-10 C00 to D48 (including breast cancer death – C50, and cancer of trachea, bronchus and lung – C33–C34 and C39); cardiovascular-related deaths as those coded I00–I99 (including ischemic heart disease (IHD) – I20–I25; and cerebrovascular diseases – I60–I69); injuries as those coded V01-Y98 [3], [14].

### Statistical analyses

Analyses were conducted separately by gender with Cox regression models stratified by centre and age in one-year age category at baseline, using attained age as main time variable. Because comparisons of socioeconomic gradients based on categorical variables may be biased if the proportionate allocation of subjects across socioeconomic strata differs, a relative index of inequality (RIIs) was also computed, separately for each stratum of sex and centre, as a relative measure of education using the lowest educational level as the referent category (adapted from [7]). This ranked variable was computed as follows: if the lowest educational group is 20% of the population, the ranked variable is assigned a value of 0.20/2 = 0.10. If the next higher educational group is 30% of the population, it is assigned a value of 0.20+0.30/2 = 0.35, etc. We used a Cox regression model with mortality as the outcome variable and the ranked variable as the explanatory variable. The RII corresponds to the estimate obtained for the ranked variable and quantifies the assumed linear effect of the relative level of education on mortality. Thus, the RII expresses inequality within the whole socioeconomic continuum and can be interpreted as the ratio of mortality between the most educated (0^th^ percentile) and the least educated (100^th^ percentile). Because the RII takes into account the size and relative position of each educational group, it is appropriate for comparing populations with different educational distributions. (for more details, see [15]).

The cumulative mortality by sex and educational level in 5-year age bands was plotted for exploring the sex and age-effect of education on mortality within the cohort (each point was calculated by comparing people recruited within the same age-group in the study across educational levels).

Covariates such as gender (where appropriate), smoking status at recruitment (never smoker, former ≥10 years, former <10 years, former unknown, current <15 cigarettes/day, 15–24 cigarettes/day, ≥25 cigarettes/day), Body Mass Index (BMI) in 2.5 kg/m^2^ categories (<20.0 to ≥37.6), alcohol consumption in deciles of distribution of g/day, leisure physical activity (divided in sex- and centre-specific tertiles of physical activity calculated on the basis of metabolic equivalent – MET – for recreational and household physical activity, plus a category for undetermined) and fruit and vegetable consumption (g/day, as continuous variable) were included into the models in a stepwise manner to estimate the extent of variation in mortality explained by these variables.

The following models were considered (stratified by centre and age): Model 1, crude (including sex if appropriate); Model 2, additionally including smoking status; Model 3, additionally including BMI; Model 4, additionally including alcohol consumption, leisure physical activity, and fruit and vegetable intake. In models investigating lung cancer mortality a more complex set of intermediate variables was used for smoking including smoking status at recruitment, age at the start and duration of smoking (in years) as continuous variables; a linear and a quadratic term for current quantity smoked (number of cigarettes per day); and two interaction terms between duration and quantity and between age at start and duration [7]. Finally, sensitivity analyses restricted to geographical regions (Northern Europe including Norway, Sweden, and Denmark; Central Europe including Netherlands, UK, and Germany; and Southern Europe, including Span, Italy, and Greece), to never smokers (Model 4a and Table S3), and replacing BMI with waist circumference in the subsample for which this information was available were conducted for all endpoints. All statistical tests were two-sided; p-values less than 0.05 were considered statistically significant; the analysis was conducted with STATA statistical software.

## Results

Demographic characteristics of the sample and geographic distribution are shown in Tables 1 and 2. Men had a higher level of education compared to women; more educated men were less likely to be ever smokers and the pattern was reversed among women. No clear association between leisure physical activity and education level among men, and an inverse association among women, were evident. BMI was strongly inversely associated with education level among women, and to a lesser extent among men.

The cumulative mortality proportion by sex and RII in 5-year age bands is plotted in Figure 1. Men experienced higher mortality in each age group and for each educational level: 19% of men with no or primary education recruited after age 65 had died at the end of the follow-up period, compared with 7% of women with a university degree recruited at the same age. Differences in mortality by educational level across age groups are more pronounced in men than in women.

In Model 1, the risk of death was significantly lower with increasing educational level; compared to the subjects with no or primary education, those with a technical/professional education had their risk of mortality lowered by about 15% (HR 0.84, 95% C.I. 0.79–0.88); those with a secondary degree lowered by about 20% (HR 0.81, 95%C.I. 0.76–0.85); and those with a university degree lowered by more than 30% (HR 0.69, 95% C.I. 0.66–0.73). These risk reduction estimates were stronger among men than women, but in both cases highly statistically significant (Table 3). When considering the Relative Index of Inequality (RII), mortality among men with the highest education is lowered by 43% compared to men with the least education level (RII_Model I_ HR = 0.57, 95% C.I. 0.52–0.61); the corresponding figure in women is 29% (RII_Model I_ HR = 0.71, 95% C.I. 0.64–0.78) (Table 3).

The risk reduction conferred by educational level on total mortality was substantially attenuated by the introduction of smoking into the model (RII_Model 2_ HR = 0.69, 95% C.I. 0.65–0.73), and additionally lowered, but to a lesser degree, after the introduction of BMI (RII_Model 3_ HR = 0.71, 95% C.I. 0.67–0.76), and additional explanatory variables (RII_Model 4_ HR = 0.75, 95% C.I. 0.70–0.80) (Table 3). These effects were consistent in men and women, and when considering absolute educational levels (Table 3, Table S1 and S2). When the same analysis was performed in never smokers, the association between educational level and total mortality among women disappeared, mainly due to the contribution of BMI, while it remained in men with a similar magnitude (Table 3, and Table S3).

Social inequalities were highly statistically significant for all causes of death examined in men except cerebrovascular mortality which was attenuated by alcohol and fruit and vegetable consumption (RII_Model4_ HR = 0.72, 95% C.I. 0.47–1.09). Inequalities were particularly high for lung cancer (RII_Model4_ HR = 0.47, 95% C.I. 0.31–0.71) and IHD mortality (RII_Model4_ HR = 0.58, 95% C.I. 0.46–0.71) (Table 4). In women, social inequalities were less strong compared to men, but statistically significant for all causes of death except for cancer-related mortality (RII_Model4_ HR = 0.99, 95% C.I. 0.86–1.14, including lung cancer mortality RII_Model4_ HR = 0.86, 95% C.I. 0.53–1.39 and breast cancer mortality RII_Model4_ HR = 1.03, 95% C.I. 0.72–1.46); and injuries (RII_Model4_ HR = 1.19, 95% C.I. 0.61–2.30) (Table 4). While social inequalities in lung cancer mortality in women seem to be explained away by smoking patterns, total cancer mortality is not associated with social inequalities even in crude models (RII_Model1_ HR = 0.93, 95% C.I. 0.80–1.07) The association with cardiovascular mortality was highly significant in both men (RII_Model4_ HR = 0.65, 95% C.I. 0.56–0.76) and women (RII_Model4_ HR = 0.62, 95% C.I. 0.49–0.78), of comparable magnitude and only partially attenuated by smoking, BMI, and alcohol and fruit and vegetable consumption (Table 4).

Social inequalities were consistent among women across the European regions (RII_Model 4_ HR = 0.84, 95% C.I. 0.72–0.97 in Northern, RII_Model 4_ HR = 0.84, 95% C.I. 0.71–0.98 in Central, and RII_Model 4_ HR = 0.87, 95% C.I. 0.66–1.16 in Southern European regions), while in men they showed an increasing trend going from North to South (RII_Model 4_ HR = 0.74, 95% C.I. 0.66–0.84 in Northern, RII_Model 4_ HR = 0.68, 95% C.I. 0.59–0.78 in Central, and RII_Model 4_ HR = 0.66, 95% C.I. 0.52–0.82 in Southern European regions) (Figure 2).

Among never smokers, the same associations appear somewhat reduced in magnitude among women and the contribution of BMI explains away the association with total mortality; this is consistent with recently published findings in a cohort of never smoker Scottish women (Table S3) [16]. For cause-specific mortality, although the sensitivity analysis in never smokers was largely underpowered to detect significant associations, in all cases it confirmed the direction of the association observed in the entire cohort with the only exceptions of lung cancer and IHD mortality in women, and injuries. The sensitivity analysis replacing BMI with waist circumference did not substantially change results (data not shown).

## Discussion

The present analysis is based on a large prospective study analysing individual-level socioeconomic positions (defined by education level) and a variety of explanatory variables of subjects residing in 9 European countries, and shows a strong inverse association between socioeconomic position and total mortality. The present data are remarkably consistent with those observed previously in other studies, especially in women [3]. Total mortality would be reduced by 23% in men and 16% in women by levelling the risk pattern associated to the lowest socio-economic strata to that of the highest levels. Notably, 29% of cardiovascular deaths among men and 34% among women could also be saved if everyone would have shared the risk pattern of those who studied beyond primary school.

An interesting phenomenon is suggested: mortality disparities by SES start at a young age and tend to amplify at older ages, in particular in men; this is consistent with a predominant role played by chronic degenerative diseases [17], [18]. Using educational level as proxy of socio-economic status has the double advantage of being stable over the lifespan, and to be easily and accurately recorded allowing cross-countries comparisons. However, patterns observed in 1992–1998 might be different from those observed 10–15 years later; in particular the relative contribution of smoking is likely to have increased over time, given the widening in social inequalities in smoking prevalence observed in particular in women [19]. Also, the relative contribution of obesity might have increased over time due to the obesity epidemic observed in many countries, including the European ones [20].

Educational inequalities among men are associated with all causes of death; among women, cancer-related mortality and breast cancer mortality do not appear to be associated with educational level. Conversely, educational level greatly contributes to predict cardiovascular death among both men and women, after accounting for possible explanatory variables. In some cases the inverse effect of higher educational level on cause-specific mortality is considerably attenuated (although still highly significant) after taking into account specific explanatory variables (i.e. IHD mortality among women). The effect of the absolute education level and that of the ranked educational variable used for computing the RII are consistent in terms of direction across all figures presented, suggesting that the distribution of educational levels in each centre does not affect the main effect of education on each of the outcome measures considered.

A substantial part of the observed inequalities in men and in women is eliminated after removing the effect of smoking and, to a lesser extent, BMI and other variables (alcohol consumption, physical activity and fruit and vegetables intake). Cigarette smoking contributes to health inequalities more markedly in men than in women, and specifically for cancer-related causes of death, in addition to, as one would expect, lung cancer in both genders. Conversely, the other lifestyle risk factors, and particularly alcohol consumption, physical activity and fruit and vegetable intake, greatly contribute in reducing inequalities for all cardiovascular causes of death analysed in both genders. This pattern of contribution of lifestyle factors in health disparities is consistent with some of the previous findings observed in single countries, both in the US [2] and Europe. The Whitehall study [21] found a comparable effect of explanatory variables, although the authors observed that lifestyle risk factors explain away the association with total mortality (but not cardiovascular mortality) if measured more than once during follow-up. The results of the GLOBE study in the Netherlands [22] and of the Hunt Study [23], made on geographically restricted and culturally homogenous populations, are also consistent with the present findings. Conversely, other studies claim that the association between socio-economic status and cardiovascular mortality [8] is explained away by intermediate variables, although the determinants of the unequal distributions of these factors according to socio-economic level within populations remain to be explained [8], [24]. These findings suggest a strong role of lifestyle characteristics in explaining a substantial portion of social inequalities in mortality, although probably not the entire effect, in particular for cardiovascular mortality. This has several potential explanations: a) unmeasured impact of considered variables which may explain the remaining inequalities in mortality (including material factors and health care factors in addition to psychosocial factors); b) non-linear interactions among risk factors; c) additional unmeasured variables, e.g. within a psycho-social causal model. Notably, a significant contribution to social inequalities in mortality could come from occupational factors implying exposure to many different toxicants associated mainly with cancer mortality, as some of us have already shown for lung cancer [25]. The biological pathway linking socio-economic status and mortality could be further explored using selected biomarkers; a recent study showed an inverse association between socio-economic status and age-related telomere attrition, suggesting a process of accelerated ageing among most deprived [26].

Overall, the socio-economic inequalities observed in the EPIC cohort tend to be stronger among men than among women, consistently with previous results [27]. This might be at least partly due to an uneven distribution of residual confounding effect of smoking and physical activity by educational level among men and women [28]. Also, the educational level of women might not always reflect their real SES position: social position of women can differentially impact the extent to which the socio-economic status of women is influenced by that of their husbands. Finally, some of the gender difference observed in cancer-related mortality might be due to the relative contribution of specific cancer sites: breast cancer, the most common cancer among women, tends to be more prevalent among higher social strata [29], [30] (whereas survival after breast cancer is better in advantaged groups [31]–[33]), while lung cancer, the most common cancer among men, is strongly associated with lower educational level in incidence [7], and in survival [17], [31]. The reduction in social inequalities among women from Northern to Sothern European countries suggests that education is a progressively less accurate proxy for socio-economic position going from North to South in Europe.

The sensitivity analysis carried out on never smokers attenuates the corresponding estimates of social inequalities in the whole cohort, with differences more pronounced in women than in men. The reason underlying this phenomenon could be a higher contribution of unmeasured variables which may explain the remaining inequalities in mortality (i.e. material, health care factors in addition to psychosocial factors) in women compared to men, or more simply driven by lower number of cases, in particular in the higher educational categories, among women.

A limitation of this analysis is the use of mortality data which combines the effect of disease incidence, access to treatment, and survival. Some of the observed inequalities might be due, at least in part, to a disparity in survival after disease incidence or to an uneven distribution of more lethal diseases. Although this does not change the final outcome, we should be cautious when transposing these figures to incidence data; determinants of prolonged survival might not be the same for disease incidence, and the distribution of participants in screening programmes is strongly associated with survival and with socioeconomic status [34]. In spite of such limitations, we believe that previous results and the present study should draw attention of policy-makers to the need to seriously address the impact of low SES on total and cause-specific mortality.

In conclusion, this study reports substantial social inequalities in mortality among European men and women which cannot be fully explained away by accounting for known common risk factors for chronic diseases. In particular, social inequalities remain unexplained for cardiovascular disease mortality in both genders and for cancer mortality in men. Unravelling specific factors and mechanisms explaining these associations and thus informing the development of prevention strategies ought to be one of the priorities of the public health sector.

## Supporting Information

Table S1 **Cox regression-derived Hazard Ratios (HR) for specific causes of mortality across education levels in men.**
(DOCX)

Table S2 **Cox regression-derived Hazard Ratios (HR) for specific causes of mortality across education levels in women.**
(DOCX)

Table S3 **Cox regression-derived Hazard Ratios (HR) for total mortality across educational levels and across Relative Inequality Index (RII) in crude (Model 1) and adjusted (Model 3 and 4) models in never smokers only.**
(DOCX)
