# Supplementary material for: Social Inequalities and Mortality in Europe – Results from a Large Multi-National Cohort
Source: PLoS One. 2012 Jul 25;7(7):e39013. doi: 10.1371/journal.pone.0039013 (PMC3405077; doi:10.1371/journal.pone.0039013)
Supplement: Table S1 — Cox regression-derived Hazard Ratios (HR) for specific causes of mortality across education levels in men. (DOCX) [file pone.0039013.s001.docx]

**Table S1:** Cox regression-derived Hazard Ratios (HR) for specific causes of mortality across education levels in men

| **Cancer-related mortality** | | | | | **Model 1 HR*** | **95% C.I.** | **p-value** | **Model 2 HR†** | | **95% C.I.** | **p-value** | | **Model 3 HR‡** | **95% C.I.** | **p-value** | | **Model 4 HR^**^** | **95% C.I.** | **p-value** | **Never smokers** | **Model 4a HR‡‡** | **95% C.I.** | **p-value** |  |  |
| --- | --- | --- | --- | --- | --- | --- | --- | --- | --- | --- | --- | --- | --- | --- | --- | --- | --- | --- | --- | --- | --- | --- | --- | --- | --- |
| **Cancer-related mortality** | | | | |  |  |  |  | |  |  | |  |  |  | |  |  |  |  |  |  |  |  |  |
| None/primary | | | 1,558 (3.1) | | Ref. | - | - | Ref. | | - | - | | Ref. | - | - | | Ref. | - | - | 228 (2.1) | Ref. | - | - |  |  |
| Technical | | | 731 (2.2) | | 0.91 | 0.83-1.00 | 0.044 | 0.94 | | 0.85-1.03 | 0.168 | | 0.93 | 0.85-1.03 | 0.150 | | 0.94 | 0.85-1.03 | 0.189 | 134 (1.4) | 0.91 | 0.73-1.14 | 0.428 |  |  |
| Secondary | | | 286 (1.6) | | 0.87 | 0.76-0.99 | 0.034 | 0.90 | | 0.79-1.03 | 0.131 | | 0.90 | 0.79-1.03 | 0.115 | | 0.91 | 0.80-1.04 | 0.151 | 58 (0.9) | 0.84 | 0.62-1.13 | 0.247 |  |  |
| University | | | 497 (1.5) | | 0.73 | 0.65-0.81 | <0.001 | 0.80 | | 0.72-0.89 | <0.001 | | 0.79 | 0.71-0.88 | <0.001 | | 0.80 | 0.72-0.89 | <0.001 | 133 (1.0) | 0.84 | 0.66-1.06 | 0.148 |  |  |
|  | |  |  | |  | Trend | <0.001 |  | | Trend | <0.001 | |  | Trend | <0.001 | |  | Trend | <0.001 |  |  | Trend | 0.114 |  |  |
| **Lung cancer death**†† | | | | |  |  |  |  | |  |  | |  |  |  | |  |  |  |  |  |  |  |  |  |
| None/primary | | | 445 (0.9) | | Ref. | - | - | Ref. | | - | - | | Ref. | - | - | | Ref. | - | - | 15 (0.1) | Ref. | - | - |  |  |
| Technical | | | 170 (0.5) | | 0.76 | 0.62-0.91 | 0.004 | 0.78 | | 0.60-1.00 | 0.056 | | 0.76 | 0.59-0.98 | 0.034 | | 0.76 | 0.59-0.98 | 0.038 | 7 (0.1) | 1.12 | 0.40-3.17 | 0.826 |  |  |
| Secondary | | | 58 (0.3) | | 0.62 | 0.47-0.83 | 0.001 | 0.96 | | 0068-1.34 | 0.799 | | 0.91 | 0.65-1.27 | 0.555 | | 0.92 | 0.65-1.29 | 0.618 | 2 (0.0) | 0.86 | 0.18-4.20 | 0.851 |  |  |
| University | | | 82 (0.3) | | 0.39 | 0.31-0.50 | <0.001 | 0.49 | | 0.34-0.68 | <0.001 | | 0.47 | 0.33-0.66 | <0.001 | | 0.48 | 0.34-0.68 | <0.001 | 3 (0.0) | 0.33 | 0.08-1.29 | 0.111 |  |  |
|  | |  |  | |  | Trend | <0.001 |  | | Trend | <0.001 | |  | Trend | <0.001 | |  | Trend | <0.001 |  |  | Trend | 0.104 |  |  |
| **All cardiovascular death** | | | | | |  |  |  |  |  | | |  |  |  | |  |  |  |  |  |  |  |  | |
| None/primary | | | | | 1,493 (3.0) | Ref. | - | - | Ref. | - | | | - | Ref. | - | | - | Ref. | - | - | 267 (2.0) | Ref. | - | - | |
| Technical | | | | | 608 (1.8) | 0.82 | 0.74-0.91 | <0.001 | 0.84 | 0.76-0.93 | | | 0.001 | 0.86 | 0.78-0.95 | | 0.004 | 0.89 | 0.81-0.99 | 0.031 | 114 (1.2) | 1.01 | 0.79-1.28 | 0.947 | |
| Secondary | | | | | 211 (1.2) | 0.72 | 0.62-0.83 | <0.001 | 0.74 | 0.64-0.86 | | | <0.001 | 0.77 | 0.66-0.89 | | 0.001 | 0.82 | 0.70-0.95 | 0.007 | 39 (0.6) | 0.78 | 0.55-1.11 | 0.163 | |
| University | | | | | 351 (1.1) | 0.58 | 0.51-0.66 | **<0.001** | 0.63 | 0.56-0.72 | | | <0.001 | 0.66 | 0.59-0.75 | | <0.001 | 0.71 | 0.63-0.81 | <0.001 | 70 (0.6) | 0.68 | 0.51-0.90 | 0.008 | |
|  | |  | | |  |  | Trend | **<0.001** |  | Trend | | | <0.001 |  | Trend | | <0.001 |  | Trend | <0.001 |  |  | Trend | 0.005 | |

**Table S1 Continued:** Cox regression-derived Hazard Ratios (HR) for specific causes of mortality across education levels in men

| **IHD death** | | | |  | |  | |  |  | |  | | |  |  | |  | | | |  |  |  |  |  |  | |  | | |  | | | | |
| --- | --- | --- | --- | --- | --- | --- | --- | --- | --- | --- | --- | --- | --- | --- | --- | --- | --- | --- | --- | --- | --- | --- | --- | --- | --- | --- | --- | --- | --- | --- | --- | --- | --- | --- | --- |
| None/primary | | | 832 (1.7) | Ref. | | - | | - | Ref. | | - | | | - | Ref. | | - | | | | - | Ref. | - | - | 145 (1.1) | Ref. | | - | | | - | | | | |
| Technical | | | 347 (1.0) | 0.80 | | 0.70-0.91 | | 0.001 | 0.82 | | 0.72-0.94 | | | 0.004 | 0.84 | | 0.73-0.96 | | | | 0.009 | 0.87 | 0.76-1.00 | 0.047 | 70 (0.7) | 1.08 | | 0.79-1.47 | | | 0.632 | | | | |
| Secondary | | | 123 (0.7) | 0.71 | | 0.58-0.86 | | 0.001 | 0.74 | | 0.61-0.90 | | | 0.002 | 0.76 | | 0.63-0.93 | | | | 0.007 | 0.81 | 0.67-0.99 | 0.040 | 23 (0.4) | 0.82 | | 0.52-1.30 | | | 0.397 | | | | |
| University | | | 169 (0.5) | 0.50 | | 0.42-0.60 | | <0.001 | 0.54 | | 0.46-0.65 | | | <0.001 | 0.57 | | 0.48-0.68 | | | | <0.001 | 0.62 | 0.52-0.74 | <0.001 | 37 (0.3) | 0.66 | | 0.45-0.98 | | | 0.039 | | | | |
|  |  | |  |  | | Trend | | <0.001 |  | | Trend | | | <0.001 |  | | Trend | | | | <0.001 |  | Trend | <0.001 |  |  | | Trend | | | 0.034 | | | | |
| **Cerebrovascular death** | | | |  | |  | |  |  | |  | | |  |  | |  | | | |  |  |  |  |  |  | |  | | |  | | | | |
| None/primary | | | 242 (0.5) | Ref. | | - | | - | Ref. | | - | | | - | Ref. | | - | | | | - | Ref. | - | - | 59 (0.4) | Ref. | | - | | | - | | | | |
| Technical | | | 83 (0.3) | 0.85 | | 0.65-1.12 | | 0.244 | 0.86 | | 0.66-1.13 | | | 0.293 | 0.87 | | 0.66-1.14 | | | | 0.316 | 0.91 | 0.69-1.20 | 0.505 | 20 (0.2) | 0.89 | | 0.51-1.56 | | | 0.682 | | | | |
| Secondary | | | 30 (0.2) | 0.72 | | 0.49-1.07 | | 0.106 | 0.74 | | 0.50-1.10 | | | 0.141 | 0.75 | | 0.51-1.11 | | | | 0.154 | 0.80 | 0.54-1.19 | 0.276 | 8 (0.1) | 0.70 | | 0.32-1.54 | | | 0.376 | | | | |
| University | | | 52 (0.2) | 0.68 | | 0.49-0.94 | | 0.018 | 0.72 | | 0.52-0.99 | | | 0.044 | 0.73 | | 0.53-1.01 | | | | 0.054 | 0.78 | 0.57-1.09 | 0.146 | 12 (0.1) | 0.61 | | 0.31-1.20 | | | 0.149 | | | | |
|  |  | |  |  | | Trend | | 0.008 |  | | Trend | | | 0.022 |  | | Trend | | | | 0.028 |  | Trend | 0.098 |  |  | | Trend | | | 0.117 | | | | |
| **Injuries** | | | |  |  | |  | |  |  | |  | | |  | | |  | |  | |  |  |  |  |  | | |  | | |  | |  |  |
| None/primary | | 156 (0.3) | | Ref. | | - | | - | Ref. | | - | | - | | Ref. | - | | | - | | | Ref. | - | - | 36 (0.3) | | Ref. | | | - | | | - | |  |
| Technical | | 64 (0.2) | | 0.79 | | 0.58-1.09 | | 0.148 | 0.81 | | 0.59-1.10 | | 0.178 | | 0.80 | 0.59-1.01 | | | 0.174 | | | 0.81 | 0.59-1.11 | 0.190 | 18 (0.2) | | 1.03 | | | 0.55-1.93 | | | 0.921 | |  |
| Secondary | | 30 (0.2) | | 0.64 | | 0.42-0.98 | | 0.039 | 0.66 | | 0.43-1.00 | | 0.051 | | 0.66 | 0.43-1.00 | | | 0.049 | | | 0.66 | 0.43-1.01 | 0.054 | 8 (0.1) | | 0.61 | | | 0.26-1.45 | | | 0.265 | |  |
| University | | 51 (0.2) | | 0.72 | | 0.51-1.01 | | 0.056 | 0.76 | | 0.55-1.07 | | 0.119 | | 0.76 | 0.54-1.06 | | | 0.110 | | | 0.77 | 0.55-1.08 | 0.128 | 19 (0.2) | | 1.00 | | | 0.53-1.88 | | | 0.990 | |  |
|  |  |  | |  | | Trend | | 0.024 |  | | Trend | | 0.054 | |  | Trend | | | 0.049 | | |  | Trend | 0.059 |  | |  | | | Trend | | | 0.768 | |  |

* stratified by centre of recruitment and age; †including smoking status at recruitment (never smoker, former smoker ≥10 years, former smoker < 10 years, former smoker unknown, current smoker <15 cigarettes/day, 15-24 cigarettes/day, ≥25 cigarettes/day) and stratified by centre of recruitment; ‡ including smoking status at recruitment (as in †) and BMI in 2.5 kg/m^2^ categories (<20.0; 20.1-22.5; 22.6-25.0; 25.1-22.5; 22.6-30.0; 30.1-32.5; 32.6-35.0; 35.1-37.5; ≥37.6) and stratified by centre of recruitment; ** including smoking status at recruitment and BMI (as in ‡) and alcohol consumption at recruitment (g/day, in deciles of distribution), leisure physical activity (inactive, moderately active, active, and unknown), and fruit and vegetables consumption; ††models including smoking are adjusted for smoking status at recruitment as a categorical variable (never, current, or former smoker); age at the start of, and duration of, smoking (in years) as continuous variables; a linear and a quadratic term for current quantity smoked (number of cigarettes per day); and two interaction terms between duration and quantity and between age at start and duration; ‡‡ model as in **, in never smoker only
