# Supplementary material for: Social Inequalities and Mortality in Europe – Results from a Large Multi-National Cohort
Source: PLoS One. 2012 Jul 25;7(7):e39013. doi: 10.1371/journal.pone.0039013 (PMC3405077; doi:10.1371/journal.pone.0039013)
Supplement: Table S2 — Cox regression-derived Hazard Ratios (HR) for specific causes of mortality across education levels in women. (DOCX) [file pone.0039013.s002.docx]

**Table S2:** Cox regression-derived Hazard Ratios (HR) for specific causes of mortality across education levels in women

| **Cancer-related mortality** | | | | **Model 1 HR*** | **95% C.I.** | **p-value** | **Model 2 HR^** | | **95% C.I.** | | **p-value** | **Model 3 HR~** | **95% C.I.** | **p-value** | | **Model 4 HR†** | **95% C.I.** | **p-value** |  | | **Model 4a HR‡‡** | **95% C.I.** | **p-value** | |  |  |
| --- | --- | --- | --- | --- | --- | --- | --- | --- | --- | --- | --- | --- | --- | --- | --- | --- | --- | --- | --- | --- | --- | --- | --- | --- | --- | --- |
| None/primary | | | 1,552 (1.7) | Ref. | - | - | Ref. | | - | | - | Ref. | - | - | | Ref. | - | - | 825 (1.5) | | Ref. | - | - | |  |  |
| Technical | | | 854 (1.3) | 0.95 | 0.87-1.05 | 0.316 | 0.97 | | 0.88-1.06 | | 0.480 | 0.98 | 0.89-1.07 | 0.664 | | 0.99 | 0.91-1.09 | 0.908 | 368 (1.2) | | 1.08 | 0.94-1.24 | 0.282 | |  |  |
| Secondary | | | 449 (1.1) | 1.03 | 0.92-1.16 | 0.580 | 1.03 | | 0.92-1.16 | | 0.559 | 1.06 | 0.94-1.18 | 0.352 | | 1.08 | 0.96-1.21 | 0.216 | 181 (1.0) | | 1.20 | 1.01-1.43 | 0.044 | |  |  |
| University | | | 386 (1.0) | 0.89 | 0.79-1.00 | 0.051 | 0.91 | | 0.81-1.03 | | 0.129 | 0.94 | 0.83-1.06 | 0.291 | | 0.96 | 0.85-1.08 | 0.497 | 160 (0.9) | | 0.98 | 0.82-1.18 | 0.843 | |  |  |
|  |  | |  |  | Trend | 0.173 |  | | Trend | | 0.139 |  | Trend | 0.631 | |  | Trend | 0.580 |  | |  | Trend | 0.953 | |  |  |
| **Breast cancer death** | | | |  |  |  |  | |  | |  |  |  |  | |  |  |  |  | |  |  |  | |  |  |
| None/primary | | | 247 (0.2) | Ref. |  |  | Ref. | | - | |  | Ref. | - |  | | Ref. | - |  | 138 (0.3) | | Ref. | - | - | |  |  |
| Technical | | | 142 (0.2) | 0.88 | 0.71-1.11 | 0.281 | 0.89 | | 0.71-1.09 | | 0.289 | 0.93 | 0.74-1.16 | 0.514 | | 0.92 | 0.74-1.16 | 0.497 | 78 (0.3) | | 1.24 | 0.89-1.72 | 0.188 | |  |  |
| Secondary | | | 88 (0.2) | 1.08 | 0.83-1.41 | 0.556 | 1.09 | | 0.83-1.42 | | 0.548 | 1.16 | 0.89-1.52 | 0.268 | | 1.15 | 0.87-1.50 | 0.326 | 47 (0.3) | | 1.64 | 1.12-2.40 | 0.011 | |  |  |
| University | | | 76 (0.1) | 0.87 | 0.66-1.15 | 0.331 | 0.88 | | 0.67-1.16 | | 0.358 | 0.97 | 0.73-1.28 | 0.836 | | 0.95 | 0.71-1.26 | 0.712 | 35 (0.2) | | 1.10 | 0.72-1.66 | 0.660 | |  |  |
|  |  | |  |  | Trend | 0.614 |  | | Trend | | 0.648 |  | Trend | 0.758 | |  | Trend | 0.904 |  | |  | trend | 0.223 | |  |  |
| **Lung cancer death‡** | | | |  |  |  |  | |  | |  |  |  |  | |  |  |  |  | |  |  |  | |  |  |
| None/primary | | | 215 (0.2) | Ref. | - | - | Ref. | | - | | - | Ref. | - | - | | Ref. | - | - | 52 (0.1) | | Ref. | - | - | |  |  |
| Technical | | | 129 (0.2) | 0.84 | 0.66-1.05 | 0.131 | 0.90 | | 0.67-1.21 | | 0.474 | 0.88 | 0.66-1.18 | 0.402 | | 0.91 | 0.68-1.23 | 0.555 | 16 (0.1) | | 0.82 | 0.43-1.55 | 0.535 | |  |  |
| Secondary | | | 64 (0.2) | 0.84 | 0.63-1.14 | 0.264 | 0.99 | | 0.68-1.44 | | 0.964 | 0.96 | 0.66-1.39 | 0.817 | | 1.03 | 0.70-1.50 | 0.887 | 6 (0.0) | | 0.73 | 0.30-1.80 | 0.493 | |  |  |
| University | | | 45 (0.1) | 0.64 | 0.46-0.87 | 0.008 | 0.75 | | 0.48-1.17 | | 0.206 | 0.71 | 0.45-1.12 | 0.146 | | 0.79 | 0.50-1.25 | 0.319 | 5 (0.0) | | 0.52 | 0.20-1.38 | 0.193 | |  |  |
|  |  | |  |  | Trend | 0.008 |  | | Trend | | 0.304 |  | Trend | 0.205 | |  | Trend | 0.468 |  | |  | Trend | 0.162 | |  |  |
| **All cardiovascular death** | | | | |  |  |  |  |  | |  | |  |  | |  |  |  |  | |  |  |  | |  | |
| None/primary | | | 924 (1.0) | | Ref. | - | - | Ref. | - | | - | | Ref. | - | | - | Ref. | - | - | | 500 (0.9) | Ref. | - | | - | |
| Technical | | | 325 (0.5) | | 0.76 | 0.66-0.87 | <0.001 | 0.78 | 0.68-0.89 | | <0.001 | | 0.81 | 0.70-0.93 | | 0.002 | 0.84 | 0.74-0.97 | 0.016 | | 126 (0.4) | 0.96 | 0.77-1.20 | | 0.716 | |
| Secondary | | | 139 (0.3) | | 0.67 | 0.56-0.82 | <0.001 | 0.67 | 0.55-0.81 | | <0.001 | | 0.71 | 0.58-0.86 | | <0.001 | 0.75 | 0.62-0.91 | 0.003 | | 46 (0.3) | 0.80 | 0.58-1.11 | | 0.191 | |
| University | | | 100 (0.3) | | 0.55 | 0.44-0.68 | **<0.001** | 0.57 | 0.46-0.70 | | <0.001 | | 0.61 | 0.49-0.76 | | <0.001 | 0.66 | 0.53-0.82 | <0.001 | | 38 (0.2) | 0.71 | 0.50-1.01 | | 0.059 | |
|  | |  |  | |  | Trend | **<0.001** |  | Trend | | <0.001 | |  | Trend | | <0.001 |  | Trend | <0.001 | |  |  | Trend | | 0.037 | |

**Table S2 Continued:** Cox regression-derived Hazard Ratios (HR) for specific causes of mortality across education levels in women

| **IHD death** | | | |  |  | | |  | |  |  | | |  |  | |  | |  | |  |  | |  | | |  |  | |  | |  |
| --- | --- | --- | --- | --- | --- | --- | --- | --- | --- | --- | --- | --- | --- | --- | --- | --- | --- | --- | --- | --- | --- | --- | --- | --- | --- | --- | --- | --- | --- | --- | --- | --- |
| None/primary | | 339 (0.4) | | Ref. | - | | | - | | Ref. | - | | | - | Ref. | | - | | - | | Ref. | - | | - | | | 162 (0.3) | Ref. | | - | | - |
| Technical | | 122 (0.2) | | 0.79 | 0.63-0.99 | | | 0.039 | | 0.81 | 0.65-1.01 | | | 0.061 | 0.85 | | 0.68-1.06 | | 0.154 | | 0.92 | 0.74-1.16 | | 0.494 | | | 47 (0.2) | 1.22 | | 0.84-1.77 | | 0.291 |
| Secondary | | 39 (0.1) | | 0.56 | 0.39-0.80 | | | 0.001 | | 0.54 | 0.38-0.77 | | | 0.001 | 0.59 | | 0.42-0.85 | | 0.004 | | 0.67 | 0.47-0.95 | | 0.025 | | | 13 (0.1) | 0.91 | | 0.50-1.66 | | 0.764 |
| University | | 33 (0.1) | | 0.49 | 0.34-0.71 | | | <0.001 | | 0.51 | 0.35-0.74 | | | <0.001 | 0.57 | | 0.40-0.83 | | 0.003 | | 0.66 | 0.46-0.97 | | 0.032 | | | 12 (0.1) | 0.83 | | 0.45-1.54 | | 0.560 |
|  |  |  | |  | Trend | | | <0.001 | |  | Trend | | | <0.001 |  | | Trend | | <0.001 | |  | Trend | | 0.006 | | |  |  | | Trend | | 0.686 |
| **Cerebrovascular death** | | | |  | |  | |  | |  |  | | |  |  | |  | |  | |  |  | |  | | |  |  | |  | |  |
| None/primary | | 278 (0.3) | | Ref. | | - | | - | | Ref. | - | | | - | Ref. | | - | | - | | Ref. | - | | - | | | 172 (0.3) | Ref. | | - | | - |
| Technical | | 91 (0.1) | | 0.77 | | 0.60-1.00 | | 0.048 | | 0.78 | 0.61-1.01 | | | 0.062 | 0.79 | | 0.61-1.02 | | 0.073 | | 0.80 | 0.62-1.04 | | 0.096 | | | 36 (0.1) | 0.82 | | 0.55-1.22 | | 0.326 |
| Secondary | | 46 (0.1) | | 0.81 | | 0.58-1.13 | | 0.209 | | 0.79 | 0.57-1.11 | | | 0.174 | 0.80 | | 0.57-1.13 | | 0.205 | | 0.82 | 0.58-1.15 | | 0.251 | | | 14 (0.1) | 0.78 | | 0.44-1.40 | | 0.405 |
| University | | 33 (0.1) | | 0.65 | | 0.44-0.95 | | 0.025 | | 0.67 | 0.46-0.98 | | | 0.038 | 0.68 | | 0.46-1.00 | | 0.047 | | 0.69 | 0.47-1.02 | | 0.065 | | | 14 (0.1) | 0.82 | | 0.46-1.46 | | 0.500 |
|  |  |  | |  | | Trend | | 0.012 | |  | Trend | | | 0.015 |  | | Trend | | 0.022 | |  | Trend | | 0.036 | | |  |  | | Trend | | 0.300 |
| **Injuries** | | | |  | | |  | |  |  | |  |  | | |  | |  | |  |  | |  | |  |  | |  |  | |  | |
| None/primary | | | 86 (0.1) | Ref. | | | - | | - | Ref. | | - | - | | | Ref. | | - | | - | Ref. | | - | | - | 48 (0.1) | | Ref. | - | | - | |
| Technical | | | 27 (0.0) | 0.71 | | | 0.44-1.14 | | 0.157 | 0.70 | | 0.44-1.13 | 0.146 | | | 0.67 | | 0.42-1.08 | | 0.103 | 0.69 | | 0.43-1.11 | | 0.124 | 11 (0.0) | | 0.90 | 0.44-1.87 | | 0.782 | |
| Secondary | | | 27 (0.1) | 1.13 | | | 0.70-1.84 | | 0.620 | 1.10 | | 0.68-1.79 | 0.692 | | | 1.02 | | 0.63-1.66 | | 0.938 | 1.06 | | 0.65-1.73 | | 0.821 | 16 (0.1) | | 2.30 | 1.19-4.42 | | 0.013 | |
| University | | | 27 (0.1) | 1.16 | | | 0.72-1.86 | | 0.547 | 1.16 | | 0.72-1.86 | 0.538 | | | 1.04 | | 0.64-1.67 | | 0.877 | 1.11 | | 0.68-1.80 | | 0.676 | 11 (0.1) | | 1.73 | 0.83-3.63 | | 0.144 | |
|  |  | |  |  | | | Trend | | 0.410 |  | | Trend | 0.429 | | |  | | Trend | | 0.756 |  | | Trend | | 0.571 |  | |  | Trend | | 0.028 | |

* stratified by centre of recruitment and age; †including smoking status at recruitment (never smoker, former smoker ≥10 years, former smoker < 10 years, former smoker unknown, current smoker <15 cigarettes/day, 15-24 cigarettes/day, ≥25 cigarettes/day) and stratified by centre of recruitment; ‡ including smoking status at recruitment (as in †) and BMI in 2.5 kg/m^2^ categories (<20.0; 20.1-22.5; 22.6-25.0; 25.1-22.5; 22.6-30.0; 30.1-32.5; 32.6-35.0; 35.1-37.5; ≥37.6) and stratified by centre of recruitment; ** including smoking status at recruitment and BMI (as in ‡) and alcohol consumption at recruitment (g/day, in deciles of distribution), leisure physical activity (inactive, moderately active, active, and unknown), and fruit and vegetables consumption; ††models including smoking are adjusted for smoking status at recruitment as a categorical variable (never, current, or former smoker); age at the start of, and duration of, smoking (in years) as continuous variables; a linear and a quadratic term for current quantity smoked (number of cigarettes per day); and two interaction terms between duration and quantity and between age at start and duration; ‡‡ model as in **, in never smoker only
