# Supplementary material for: Social Inequalities and Mortality in Europe – Results from a Large Multi-National Cohort
Source: PLoS One. 2012 Jul 25;7(7):e39013. doi: 10.1371/journal.pone.0039013 (PMC3405077; doi:10.1371/journal.pone.0039013)
Supplement: Table S3 — Cox regression-derived Hazard Ratios (HR) for total mortality across educational levels and across Relative Inequality Index (RII) in crude (Model 1) and adjusted (Model 3 and 4) models in never smokers only. (DOCX) [file pone.0039013.s003.docx]

**Table S3:** Cox regression-derived Hazard Ratios (HR) for total mortality across educational levels and across Relative Inequality Index (RII) in crude (Model 1) and adjusted (Model 3 and 4) models in never smokers only.

|  | | **N** | **Mortality (%)** | **Model 1 HR*** | **95% C.I.** | **p-value** | **Model 3 HR‡** | **95% C.I.** | **p-value** | **Model 4 HR**** | **95% C.I.** | **p-value** |
| --- | --- | --- | --- | --- | --- | --- | --- | --- | --- | --- | --- | --- |
| **Men** | | **41,876** |  |  |  |  |  |  |  |  |  |  |
| Educational level | |  |  |  |  |  |  |  |  |  |  |  |
| None/primary | | 13,638 | 791 (5.8) | Ref. | - | - | Ref. | - | - | Ref. | - | - |
| Technical | | 9,638 | 378 (3.8) | 0.87 | 0.76-1.00 | 0.044 | 0.90 | 0.78-1.02 | 0.107 | 0.91 | 0.80-1.04 | 0.167 |
| Secondary | | 6,474 | 156 (2.4) | 0.80 | 0.67-0.96 | 0.018 | 0.84 | 0.70-1.01 | 0.060 | 0.86 | 0.72-1.03 | 0.111 |
| University | | 11,930 | 318 (2.7) | 0.71 | 0.62-0.82 | <0.001 | 0.76 | 0.66-0.88 | <0.001 | 0.78 | 0.67-0.90 | 0.001 |
|  |  |  |  | Trend |  | <0.001 |  | Trend | <0.001 |  | Trend | 0.001 |
| RII | |  |  | 0.62 | 0.52-0.76 | <0.001 | 0.68 | 0.56-0.83 | <0.001 | 0.71 | 0.59-0.87 | 0.001 |
| **Women** | | **122,944** |  |  |  |  |  |  |  |  |  |  |
| Educational level | |  |  |  |  |  |  |  |  |  |  |  |
| None/primary | | 55,753 | 1,749 (3.1) | Ref. | - | - | Ref. | - | - | Ref. | - | - |
| Technical | | 29,841 | 727 (2.4) | 0.98 | 0.89-1.09 | 0.739 | 1.02 | 0.92-1.12 |  | 1.03 | 0.93-1.14 | 0.532 |
| Secondary | | 18,628 | 323 (1.7) | 1.04 | 0.91-1.18 | 0.581 | 1.10 | 0.96-1.25 | 0.163 | 1.12 | 0.98-1.27 | 0.100 |
| University | | 18,722 | 262 (1.4) | 0.83 | 0.72-0.95 | 0.007 | 0.89 | 0.77-1.02 | 0.091 | 0.90 | 0.78-1.04 | 0.152 |
|  |  |  |  | Trend |  | 0.050 |  | Trend | 0.447 |  | Trend | 0.657 |
| RII | |  |  | 0.88 | 0.76-1.03 | 0.124 | 0.98 | 0.83-1.14 | 0.760 | 1.01 | 0.86-1.18 | 0.918 |

* stratified by centre of recruitment and age; **‡**including BMI in 2.5 kg/m^2^ categories (<20.0; 20.1-22.5; 22.6-25.0; 25.1-22.5; 22.6-30.0; 30.1-32.5; 32.6-35.0; 35.1-37.5; ≥37.6) and stratified by centre of recruitment; ** including BMI (as in ‡) and alcohol consumption at recruitment (g/day, in deciles of distribution), leisure physical activity (inactive, moderately active, active, and unknown), and fruit and vegetables consumption
